# A Comprehensive Review on Lipid Oxidation in Meat and Meat Products

**DOI:** 10.3390/antiox8100429

**Published:** 2019-09-25

**Authors:** Rubén Domínguez, Mirian Pateiro, Mohammed Gagaoua, Francisco J. Barba, Wangang Zhang, José M. Lorenzo

**Affiliations:** 1 Centro Tecnológico de la Carne de Galicia, Rúa Galicia Nº 4, Parque Tecnológico de Galicia, 32900 San Cibrao das Viñas, Spain; rubendominguez@ceteca.net (R.D.); mirianpateiro@ceteca.net (M.P.); 2 Food Quality and Sensory Science Department, Teagasc Ashtown Food Research Centre, Ashtown, Dublin 15, Ireland; gmber2001@yahoo.fr; 3 Preventive Medicine and Public Health, Food Science, Toxicology and Forensic Medicine Department, Faculty of Pharmacy, Universitat de València, 46100 València, Spain; francisco.barba@uv.es; 4 Key Laboratory of Meat Processing and Quality Control, Ministry of Education China, Jiangsu Collaborative Innovation Center of Meat Production and Processing, Quality and Safety Control, College of Food Science and Technology, Nanjing Agricultural University, Nanjing 210095, China; wangang.zhang@yahoo.com

**Keywords:** oxidative deterioration, hydroperoxide, volatile compounds, aldehydes, thiobarbituric acid reactive substances (TBARs), analytical methods, free radicals

## Abstract

Meat and meat products are a fundamental part of the human diet. The protein and vitamin content, as well as essential fatty acids, gives them an appropriate composition to complete the nutritional requirements. However, meat constituents are susceptible to degradation processes. Among them, the most important, after microbial deterioration, are oxidative processes, which affect lipids, pigments, proteins and vitamins. During these reactions a sensory degradation of the product occurs, causing consumer rejection. In addition, there is a nutritional loss that leads to the formation of toxic substances, so the control of oxidative processes is of vital importance for the meat industry. Nonetheless, despite lipid oxidation being widely investigated for decades, the complex reactions involved in the process, as well as the different pathways and factors that influenced them, make that lipid oxidation mechanisms have not yet been completely understood. Thus, this article reviews the fundamental mechanisms of lipid oxidation, the most important oxidative reactions, the main factors that influence lipid oxidation, and the routine methods to measure compounds derived from lipid oxidation in meat.

## 1. Introduction

Lipids are compounds of vital importance for proper human nutrition. In addition to providing energy to the biological processes of the body, lipids contain a large number of substances such as essential fatty acids or fat-soluble vitamins that can only be provided by the diet. Furthermore, lipids are responsible for many desirable characteristics of meats and meat products [1]. They influence the flavour and contribute to improving the tenderness and juiciness of meats [2]. Therefore, fat content and composition are of major importance for consumers due to their importance for meat quality and nutritional value [3].

However, lipids are susceptible to degradation. Lipid oxidation is the main non-microbial cause of quality deterioration in meat and meat products [4,5]. The degradation begins with the sacrifice of the animal and continues progressively until the final product is consumed [6]. Therefore, all intermediate processes (handling, processing and storage) must be carefully controlled in order to prevent these reactions [7] and to minimize the economic losses of the meat industry [8]. In fact, the main objective of the industry and researchers is to understand the lipid oxidation mechanisms and identify the most effective methods to control this process [2,9].

Oxidation reactions not only reduce the nutritional value of meats due to the loss of essential fatty acids and vitamins. Generally, the first change observed results in a gradual reduction of sensory quality. These include changes in colour, texture, and appearance of rancid odour and flavour, which influences consumer acceptance [1]. In addition, multiple toxic compounds are produced during lipid oxidation. Several authors conclude that one of the most important problems of lipid oxidation is the generation of harmful compounds that implicate several human pathologies, including atherosclerosis, cancer, inflammation and aging processes, among others [10,11,12]. In this regard, a study concluded that lipid hydroperoxides contribute to cell cytotoxicity and that low concentrations of hydroperoxides would exert toxic effects on cells [13]. The cholesterol oxidation products also are more dangerous for arterial cells than cholesterol and are related to coronary diseases, mutagenic activity and atherosclerosis [12]. Recent research found that aldehydes and oxysterols derived from lipid oxidation had pro-inflammatory, cytotoxic and mutagenic effects [14]. Therefore it seems clear that oxidation products are involved in the development or promotion of innumerable diseases [15,16].

Although lipid oxidation generally has negative effects on meat and meat products, in some cases it contributes to the development of pleasant aromas [17,18,19,20,21,22,23]. In fact, it is well known that the compounds derived from lipid oxidation play an important role in the development of the typical aroma of meat products during ripening or dry-cured stages, which is one of the most appreciated consumers’ attributes [6,24].

The lipid oxidation is a very complex process, which includes multiple mechanisms that interact with each other. To explain it simply, unsaturated fatty acids react with molecular oxygen via a free radical mechanism. Derived from this reaction, hydroperoxides are produced that are considered to be the first oxidation products. In contrast to other lipid-derived products, hydroperoxides are odourless and do not contribute any aroma. However, these compounds are highly unstable, so they decompose rapidly resulting in a large number of secondary compounds that include hydrocarbons, aldehydes, ketones, alcohols, esters and acids [25], which cause the appearance of off-flavours and off-odours in meat. Nonetheless, not all of these compounds are equally important in affecting the overall aroma perception. The importance of each compound in the final aroma depends on the concentration and the olfactory threshold [26]. Within all compounds derived from oxidation processes, aldehydes are considered the most important breakdown products and the largest contributors to volatile flavours in meat [27]. This aspect is related to the fact that they have a low odour threshold and that they are present in significant quantities in the products that suffered oxidation processes [24]. The predominant aldehydes derived from lipid oxidation include n-alkanals, trans-2-alkenals, 4-hydroxy-trans-2-alkenals and malondialdehyde [6]. In addition to contributing to the aroma of meat, aldehydes are also important because they react with proteins causing modifications that result in changes in nutritional and organoleptic properties [28].

Therefore, taking into account everything mentioned above, it seems clear that the shelf life of meat is determined by the moment in which the consumer is able to detect the oxidation products that impart rancidity (mainly volatile) or observe changes in meat colour [29]. The main strategy used by the meat industry to inhibit lipid oxidation is the addition of antioxidants to meat and meat products [30,31,32,33,34,35,36]. However, nowadays consumers demand more natural products, which limits the industry in their use of currently allowed synthetic antioxidants in foods, leaving manufacturers with few options [29]. In addition, consumers are becoming more aware of the unhealthy effects of a high amount of fat and saturated fatty acids in meat and meat products. Therefore, a growing interest exists in developing new products with a health concept such as a “high polyunsaturated fatty acids” or “n-3 enriched” products [37,38,39,40,41,42,43]. To do this, traditional products must be reformulated, replacing part of the animal fat with polyunsaturated fatty acids sources [38,44]. However, this has a huge effect on the shelf life and oxidative stability of meat products. In order to overcome this problem, several lines of research are booming, mainly focused on obtaining natural antioxidants that replace synthetic additives and the use of active and intelligent packaging to try to control the possible evolution of oxidation [9].

On the other hand, in spite of the innumerable researches carried out during several decades with the aim of understanding the theoretical and practical mechanisms of lipid oxidation, the knowledge of some of them is not completely clear. In fact, some reactions are missing, incorrect, or incomplete in the current understanding of lipid oxidation [45]. Thus, to design strategies to prevent lipid oxidation in meat effectively, mechanisms, reactions and the main factors involved in the process should be comprehensively understood [4,6].

This manuscript on lipid oxidation in meat and meat products will review the fundamental mechanisms of lipid oxidation, the most important oxidative reactions, the main factors that influence lipid oxidation and some of the commonly used methods to measure compounds derived from lipid oxidation in meat.

## 2. Mechanism of Lipid Oxidation 

It is well known that unsaturated fatty acids and oxygen are the components that react during the lipid oxidation process. Additionally, other components can promote or prevent oxidation reactions. Lipids can be oxidized by three main ways that include complex reactions: autoxidation, enzymatic-catalysed oxidation and photo-oxidation. Among the three mechanisms, autoxidation, which is a continuous free-radical chain reaction, is the most important process of lipid oxidation in meat [2,27,46]. The enzymatic and photo-oxidation mechanisms only differ from autoxidation in the formation of hydroperoxides, during the initiation phase [6]. The mechanism of free radicals, despite explaining many of the changes observed in meat, does not provide a detailed and complete description of the changes produced in reactants and derived products during the oxidation process. Therefore, the main challenge is to complete the scheme that can fully explain all the agents, intermediate products and reactions involved [47].

### 2.1. Lipid Autoxidation

As commented above, autoxidation is the main process by which unsaturated fatty acids and oxygen interact, and therefore produce an oxidative deterioration of meat and meat products [6,46]. Normally, the autoxidation process is usually represented as a combination of three distinct phases: The initiation in which free radicals occur, the propagation in which the number of reactive compounds is multiplied, and finally the termination in which the reactive compounds degrade or react with each other to give non-reactive compounds [27].

#### 2.1.1. Initiation

**Figure 1:**
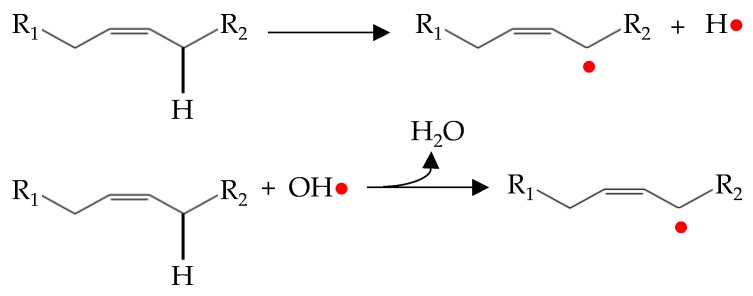
Simple representation of the initiation process of lipid oxidation.

The initiation stage of lipid oxidation is still an area of controversy and remains the subject of much research [48]. It is well known that the interaction between an unsaturated fatty acid and an oxygen molecule is not a spontaneous reaction. In fact, oxygen is in triplet electronic state while double bonds of fatty acids are in singlet electronic state. This aspect determines that they cannot react directly because of the different spin states [4,16]. In addition, the triplet oxygen also cannot convert to singlet states itself [16]. Therefore, before the reaction, the oxygen must be activated resulting in the formation of singlet oxygen (^1^O_2_) or a reactive oxygen species as hydrogen peroxide (H_2_O_2_), superoxide anion (O_2_•^−^) and hydroxyl radical (OH•) (Figure 1) [46]. It is well agreed that the activation of oxygen is mediated by a source of energy (temperature or light) and/or by the presence of catalytic compounds as transition metals [4].

**Figure 2:**
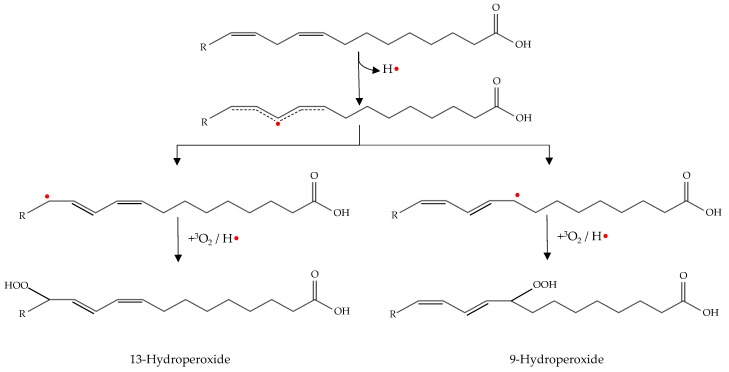
Double-bound rearrangement with production of conjugated dienes (initiation phase) and subsequent formation of hydroperoxides (propagation phase).

Initiation occurs as hydrogen is abstracted from an unsaturated fatty acid. The resulting alkyl radical tends to be stabilized by a double-bound rearrangement to form a conjugated dienes or trienes (Figure 2) [49]. 

These alkyl radicals are the first free radicals that initiate lipid oxidation [47]. Therefore, initiation is frequently attributed to the reaction of the fatty acids with active oxygen species [6,46]. In the initiation stage, a lag phase is normally observed where accumulation of lipid oxidation products is slow. This is mainly due to the slow formation of free radicals prior to hydroperoxides accumulation and that the free radicals preferentially oxidize the natural antioxidants present in meat, which protects fatty acids at the earliest stages of oxidation [29].

#### 2.1.2. Propagation

**Figure 3:**
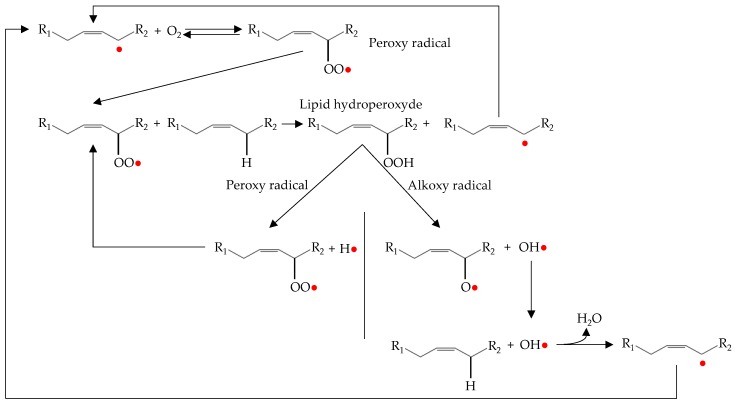
Representation of propagation and magnification processes during lipid oxidation.

The propagation phase of oxidation occurs by lipid-lipid interactions resulting in a magnification of radical formation (Figure 3). 

The alkyl radical produced during the initiation phase reacts with the molecular oxygen to form peroxy radicals (radical coupling with oxygen) [4]. The peroxy radical is highly reactive and abstract hydrogen from an adjacent lipid (atom transfer process). This process results in a hydroperoxide and alkyl radical. The new alkyl radical reacts again with molecular oxygen to form new peroxy radicals and the process is repeated again [6].

As can be seen in Figure 3, in addition to the propagation phase there is also a magnification phase. This is known as secondary initiation, in which hydroperoxides formed during propagation decompose to give rise to new hydroxyl, peroxy and alkoxy radicals. These radicals are highly reactive and can abstract a hydrogen atom from an unsaturated fatty acid. Therefore, they act as initiators of autoxidation and stimulate the lipid oxidation processes [6,8]. 

There are two main pathways for the decomposition of hydroperoxides into radicals. The first is mediated by the presence of metals. In this way, the metal ion transfers an electron to the hydroperoxide, which causes its fragmentation. Therefore, the iron present in meat (both, heme and non-heme) is an important catalyst for the decomposition of hydroperoxides, being faster in reaction with ferrous ion than with the ferric ion [46]. The second pathway involves the interaction between two hydroperoxides. In this case, it is necessary that two hydroperoxide molecules be close enough to form bimolecular associations, which are then broken by homolytic cleavage between the oxygen and the oxygen bond producing alkoxy and hydroxyl radicals [47,50]

#### 2.1.3. Termination

The termination phase consists of the reaction between radicals or with other non-radical compounds (antioxidants) to give rise to non-radical products. In the case of the reaction between two radicals, radical–radical coupling and disproportionation can occur to form a non-radical adduct. In fact, the reactions between peroxy, alkoxy and/or alkyl radicals are usually represented as follows [27,51]:

R• + R• → R–R
R• + ROO• → ROOR
RO• + RO• → ROOR
RO• + R• → ROR
ROO• + ROO• → ROOR + O_2_
2RO• + 2ROO• → 2ROOR + O_2_

In a similar way, an antioxidant compound could transfer a hydrogen atom to the radical species derived from lipid oxidation. This reaction neutralizes the lipid radical and creates a new radical from the antioxidant compound that is much less reactive [46].

**Figure 4:**
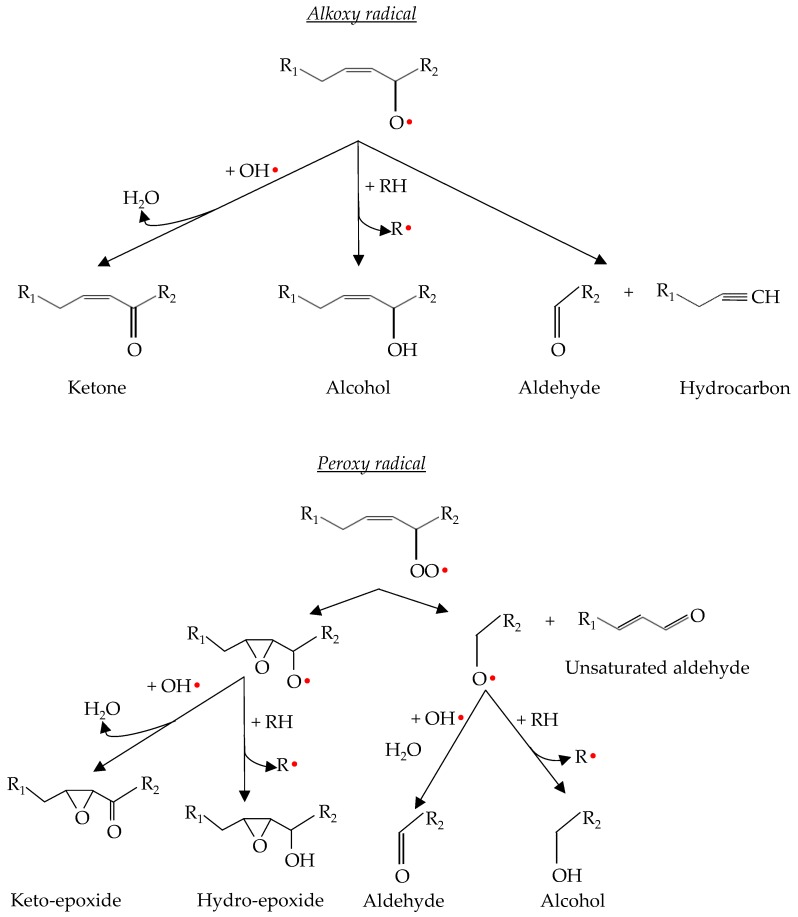
Alkoxy and peroxy radicals’ decomposition into secondary oxidation products.

Therefore, in both cases, stable or low-reactive products are formed from radicals by an atom or group transfer process. However, termination reactions are not always efficient and may lead to new reactive compounds. The mechanism that ensures termination efficiently is the decomposition of peroxy and alkoxy radicals to give rise to secondary products such as alkanes, alcohols and carbonyl compounds [46]. The process of decomposition of peroxy and alkoxy radicals is explained in Section 2.4 of the present review and is represented in Figure 4.

### 2.2. Lipid Photo-Oxidation

Usually, meat and meat products are directly exposed to light in the supermarket to be attractive to consumers. This fact promotes the photo-oxidation process, that is much faster than autoxidation [52]. Photo-oxidation is another mechanism of initiation of lipid oxidation. During this process, hydroperoxides are formed in the presence of sensitizers (as myoglobin or hemoglobin) and light. Therefore, photo-oxidation is an alternative route for the formation of hydroperoxides instead of the free radical mechanism explained in the autoxidation process [53].

The first step of photo-oxidation is the excitation of singlet sensitizer by absorbing light energy, giving rise to the excited triplet sensitizers. Then the photo-oxidation reactions could be divided into three main pathways:

In the first pathway, excited triplet sensitizers (^3^S*) react with molecular oxygen (^3^O_2_) and produce singlet oxygen (^1^O_2_) via a triplet-triplet annihilation mechanism [46]. This is the most common mechanism for the production of singlet oxygen [46]. Then, the singlet oxygen can react directly with moieties of high electron density of double bonds of unsaturated fatty acids producing a hydroperoxide without the formation of the alkyl radical [50,53]. 

On the other hand, excited sensitizer can react with triplet oxygen and produce superoxide radical anion (O_2_•^−^) by electron transfer. This reactive oxygen species could abstract hydrogen from unsaturated fatty acids and initiate the lipid oxidation. Additionally, superoxide radical anion reacts with hydrogen peroxide and produces both, hydroxyl radical and singlet oxygen (H_2_O_2_ + O_2_•^−^ → HO• + OH^−^ + ^1^O_2_), which can react directly with fatty acids and initiate lipid oxidation. This reaction is catalysed by the presence of metals [50].

Finally, the excited triplet sensitizer can also abstract hydrogen from an unsaturated fatty acid, resulting in the production of alkyl radical [50,53]. Then, this alkyl radical reacts with molecular oxygen giving rise to a peroxy radical that can abstract hydrogen from an adjacent fatty acid initiating the free radical chain reactions mechanism, as described above in the propagation phase [46].

### 2.3. Enzymatic Lipid Oxidation

In addition to the non-enzymatic mechanisms explained, there is also an enzyme-mediated mechanism that initiates lipid oxidation. As when this occurs in photo-oxidation, the main difference between enzyme-catalysed lipid oxidation and free radical initiation is the formation of hydroperoxides. 

The main enzyme involved in enzymatic oxidation is lipoxygenase. It should be noted that the amount of lipoxygenase plays an important role in the development of oxidation. It is well known that the enzymatic lipid oxidation presents an initial lag phase, which is inversely proportional to the lipoxygenase concentration [54]. Thus, the enzyme concentration determines the rate at which lipid oxidation develops, so high concentration favoured oxidative processes.

This enzyme has an active site that contains iron that must be in ferrous form for the enzyme exhibits activity [47]. The active site of enzyme abstracts a hydrogen atom from the methylene group of a polyunsaturated fatty acid to form a conjugated diene system that reacts with molecular oxygen. The peroxy radical removes hydrogen from another unsaturated fatty acid molecule and finally a conjugated hydroperoxy diene and alkyl radical is generated [6,53]. 

### 2.4. Decomposition of Hydroperoxides and Alkoxy and Peroxy Radicals

Lipid hydroperoxides are not considered harmful to food quality because they are odourless and tasteless [46]. However, hydroperoxides are unstable compounds, so they tend to decompose into their alkoxy and peroxy radicals [55]. These radicals are further degraded into secondary compounds that are responsible for sensory deterioration such as odours and flavours associated with lipid oxidation [7]. The main secondary compounds released include lipid alcohols, ketones, epoxides, aldehydes and hydrocarbons (Figure 4). Additionally, unsaturated aldehydes can be oxidized further and additional volatile products may be formed [46]. The formation of these compounds is mainly produced via α- or β-scissions reactions, and it is minimal during the initiation phase but increases exponentially during the propagation and termination phases [4,47]. 

It is important to highlight the influence of this type of reaction in lipid oxidation. As mentioned in the lipid oxidation termination processes, these reactions are expected to give rise to stable compounds. However, α- and β-scission, internal rearrangement to epoxides and disproportionation of lipid radicals are active reactions that can abstract hydrogens of unsaturated fatty acids or hydroperoxides, which promotes the propagation phase and, therefore, lipid oxidation. Thus, the reactions that take place during the decomposition of hydroperoxides and their radicals can substantially vary the lipid oxidation kinetics as well as the oxidation products [45]. 

## 3. Factors Affecting Lipid Oxidation

As stated above, lipid oxidation is a process that includes multiple mechanisms with very complex reactions and interactions between substrates and catalyst. These reactions are influenced by different factors. In fact, both intrinsic (meat composition) and extrinsic factors (processing and storage conditions) can promote or inhibit the oxidative reactions [52,53]. Therefore, the oxidative stability of meat depends on the balance of anti- and prooxidant compounds [10].

Regarding the intrinsic parameters, the fatty acid composition is one of the most important parameters, since they are the main substrate for the development of lipid oxidation. However, the content of other prooxidant compounds, such as heme-proteins, metals, prooxidant enzymes or antioxidant compounds such as vitamins, antioxidant enzymes or peptides, are determinants in the development of oxidative processes. Indirectly, factors such as muscle type, species or breeds, rearing system, anatomical location or diet received by animals, among others, affect oxidation because they significantly modify meat composition [4,8].

On the other hand, storage conditions have enormous relevance in the promotion of lipid oxidation [56,57,58]. In fact, different factors such as temperature or the presence of light and oxygen increase oxidative processes [53]. Similarly, certain processing treatments also promote oxidation. In this regard, processes such as cutting, deboning, grinding or cooking accelerate the development of lipid oxidation [27]. Generally, all processes cause disruption of the muscle membrane promoting oxidative reactions. This is related to processing liberated membrane phospholipids, increasing their contact with prooxidant components such as oxygen, enzymes or metals that promote the oxidation [4,7,27]. Additionally, the use of other ingredients (anti or prooxidants) in meat product formulations also influences the oxidation process [6]. The effect of different processes and the application of new emerging technologies to increase the meat shelf life (electric pulses, high pressures, irradiation, etc.) on the development of oxidation have been reviewed extensively [6,46,52] and therefore will not be covered in this review.

### 3.1. Meat Composition

#### 3.1.1. Fat Content and Fatty Acids Composition

The main factors that influence lipid oxidation in meat are fat content and fatty acid composition because fatty acids are the substrate of oxidation processes. In meat, lipids are organized into triglycerides and phospholipids, with low contributions of other types of lipids such as free fatty acids, cholesterol or vitamins. The amount of intramuscular fat is directly related with the number of triglycerides, because they are the reserve lipids (≈95% of meat lipids) [10], while the phospholipids represent about 500 mg/100 g of meat [59]. Therefore, an increase of intramuscular fat results in an increase in the volume of fatty cells (triglycerides), while the number of phospholipids remains constant since the number of membranes does not vary. 

Although the major lipid amounts are in triglycerides fraction, multiple researchers pointed out that phospholipids are essential in the development of lipid oxidation. There are two main explanations for this high phospholipid reactivity. First, the arrangement of lipids in membranes facilitates the propagation phase of lipid oxidation, because oxidation catalytic sites are closer [46]. Additionally, it is well known that phospholipids have a higher amount of polyunsaturated fatty acids than triglycerides [2]. Different studies carried out on fresh meat from pork and beef showed that total polyunsaturated fatty acids in neutral fraction (triglycerides) represent between 4.5–14% while this amount increases up to 37–47% in the polar lipids fraction (phospholipids) [60,61,62]. Therefore, it is clear that phospholipids are the major contributors to the development of lipid oxidation and rancidity [27,46]. To this regard, a research conducted to observe the role of phospholipids and triglycerides on malondialdehyde formation concluded that phospholipid fraction contributed about 90% of the malondialdehyde [63].

On the other hand, there is controversy about the effect that the fat amount has on meat oxidation. The contents of fat and fatty acids of different meat and meat products are shown in Table 1 and Table 2. Some authors affirm that total lipids are the major contributor to rancidity on pork [64], while other authors pointed that the fatty acids composition of fat is more important than the amount of fat [65]. In lean meat (with very low intramuscular fat), having a high percentage of phospholipids makes it very susceptible to oxidation [27]. Therefore, regarding oxidative susceptibility, the unsaturation of fat is much more important parameter than fat amount. 

**Figure 5:**
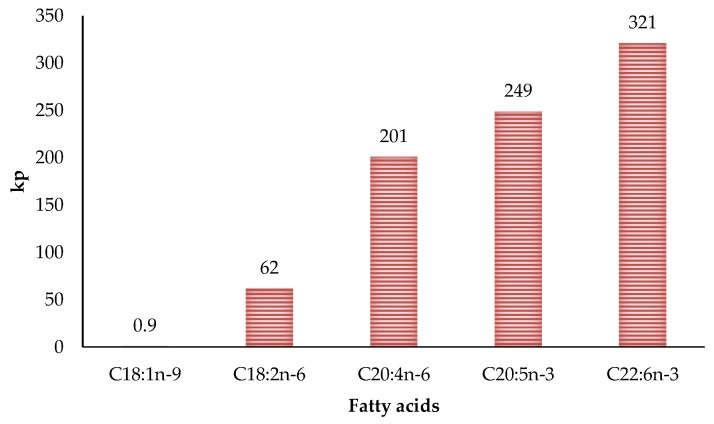
Relationship between number of double bonds and propagation rate constants (kp) of lipid oxidation.

The relationship between the degree of fat unsaturation and oxidative stability has been extensively studied for years. It is well known that oxidation susceptibility is correlated exponentially with the number of unsaturation of fatty acids. This is due to carbon-hydrogen bond dissociation energies being lower in allylic (R_1_–CH=CH–CH_2_–R_2_) and bis-allylic methylene (R_1_–CH=CH–CH_2_–CH=CH–R_2_) positions than in fatty acids lacking double bonds [46,71]. Therefore, the greater the double bonds, the greater the oxidative susceptibility due to more reaction sites [29]. These reaction sites are thermodynamically favoured position for attack by radicals in polyunsaturated fatty acids. Moreover, hydrogen atoms from the bis-allylic methylene position are around 20 times more abstractable than the allylic position [7]. Thus the difference in the propagation rate constants for fatty acids are correlated to the number of bis-allylic positions (Figure 5) [72]. This aspect is very important in poultry meat since it has a much higher degree of unsaturation than the meat of other species. In fact, different researchers found that polyunsaturated fatty acids values can reach 27.5% of the total fatty acids in turkey meat [66], 37% in chicken [73] and 65% in goose [74]. 

In addition to the number of double bonds, the fatty acids conformation also affects their oxidative stability. Some authors pointed out that *trans* isomers are significantly more stable than *cis* isomers [47,82]. The lipid oxidation also increases when fatty acid chain length is increased [83]. Furthermore, the position of the bis-allylic methylene influences the oxidative susceptibility of fatty acids. In this regard, it was reported that the oxidation rate is higher in n-3 than in n-6 polyunsaturated fatty acids [84]. 

#### 3.1.2. Cholesterol and Cholesterol Oxidation Products 

Cholesterol is an essential component of animal tissue whose chemical structure makes it susceptible to oxidation. The process of oxidation of cholesterol is similar to those which occur in other unsaturated lipids, since it is prone to react with reactive oxygen species (ROS) [85]. The double bonds between carbons 5 and 6, carbon 7 and tertiary carbons C20 and C25 are the main positions sensitive to oxidation [86]. Both the compounds resulted from the oxidation and their concentrations depend on several processing or storage factors like heat, pH, light, oxygen, oxidation time, type of buffer, water activity, the form of substrate, and the presence of unsaturated fatty acids [87,88].

Between the resulted cholesterol oxidation products (COPs) the formation of mono- or polyoxygenated compounds are included, characterized by the presence of additional polar groups [89]. The most common COPs present in meat and meat products are 7-ketocholesterol, 20α-hydroxycholesterol, 25-hydroxycholesterol, α, β-epoxycholesterol, and 7α, 7β-hydroxycholesterol [90,91,92,93]. However, the contents found for 7-ketocholesterol revealed that this is the most abundant cholesterol oxide. Concentrations between 57 to 71 µg/100 g can be found in meat and meat products [91,94], it can even be used as a marker of the total oxidative process [12].

#### 3.1.3. Heme-Proteins and Metals

The presence of metals, either in the form of heme-proteins or in free form is one of the main factors that determine the oxidative stability of meat. In fact, some authors have pointed out that the presence of metals promotes oxidation process more efficiently than temperature or light [6]. This is because transition metals such as iron or copper are potent catalysts of different processes and stages of lipid oxidation [7,28]. In animal tissues, 90% of iron occurs in the form of hemoglobin, myoglobin and low amounts of ferritin, transferrin and active sites of some enzymes. Among them, the most abundant heme-proteins present in meat are myoglobin and hemoglobin. From these heme-proteins, free iron could be generated by the destruction of heme group and release of iron [6]. Additionally, heme-protein amounts vary with many factors such as animal species, muscle type and anatomical location of muscle, therefore, the oxidative stability of meat depending on the heme-protein amounts due to lipid oxidation is concentration-dependent [4,6]. 

The system of action of metals is complex, but their role as catalysts of lipid oxidation is because they are capable of catalysing the production of reactive oxygen species. These processes are frequently explained by the Fenton reaction [4,47]. This reaction is effective when Fe^3+^ can be reduced to Fe^2+^ by reducing compounds [46] and it can involve other ions such as Cu^2+^, Ti^4+^, and Co^3+^ [95,96].

Fe^2+^ + O_2_ → Fe^3+^ + O_2_•^−^
2 O_2_•^−^ + 2 H^+^ → H_2_O_2_ + O_2_
Fe^2+^ + H_2_O_2_ → Fe^3+^ + OH^−^ + OH•

The first step of the Fenton reaction is the oxidation of ferrous (Fe^+2^) to ferric form (Fe^+3^). In this oxidation, Fe^+2^ reacts with oxygen and produces Fe^+3^ and superoxide anion (O_2_•^−^). The O_2_•^−^ can react with fatty acids to produce alkyl radicals and initiate lipid oxidation. In addition, O_2_•^−^ could be transformed into hydrogen peroxide by dismutation, which could react with Fe^+2^ to produce hydroxyl radical (OH•). In this regard, the Fenton reaction is the major pathway to produce OH•. It is likely the most reactive oxygen species and it is highly important in the initiation stage of the oxidation process [95].

In a similar way, the Fenton reaction is the main mechanism for the heme-protein oxidation [6]. In addition to the development of rancidity, the oxidation of heme-proteins results in a deterioration of meat colour during storage, which has an important effect on consumer acceptability. Several authors found a relationship between lipid and heme-protein oxidations. In fact, lipid and heme-protein oxidation in meat occurs in a concurrent manner and each process appears to enhance the other [51]. 

The oxy heme-protein oxidation process results in met heme-protein and hydroperoxyl radical (OOH•) or O_2_•^−^, which are converted to hydrogen peroxide (H_2_O_2_). Then, met hem-protein can react with H_2_O_2_ or preformed lipid hydroperoxide to produce a highly reactive ferryl heme-protein radical [6,7]. So, all of the reactive oxygen species generated during oxy heme-protein oxidation (H_2_O_2_, OOH• or O_2_•^−^) as well as the ferryl radical can abstract hydrogen from polyunsaturated fatty acid and consequently initiate lipid oxidation [51]. 

There are various conditions that could vary the rate of oxy and met heme-protein oxidation. Different studies found higher oxidation at levels lower than neutral pH [6]. This could be related to the higher iron solubility in low pH and that heme-protein oxidation is favoured at reduced pH [6]. In contrast, the prooxidant effect of ferryl heme-protein is independent of pH [97]. Therefore, ferryl heme-protein is expected to be an effective prooxidant in meat [6]. 

On the other hand, the presence of lipid oxidation products could also favour heme-protein oxidation. Aldehydes alter heme-protein redox stability, resulting in the promoted oxidation of oxy heme-protein, decreasing the met heme-protein reduction and enhancing its prooxidant activity [51]. Regarding this fact, it should be added that during the lipid oxidation a wide variety of aldehydes are released, so it would explain in part how lipid oxidation increases the oxidation of heme-proteins.

In addition to the role of Fe^+2^ (both, heme and free form) in the initiation step of lipid oxidation, it also has an important role in the propagation phase [4,52]. Fe^+2^ reacts with the preformed hydroperoxides and decomposes them into alkoxy and hydroxyl radicals, which readily react with other adjacent molecules and in the presence of oxygen magnify the propagation phase [46].

#### 3.1.4. Prooxidant Enzymes

Several enzyme systems identified in meat are capable of initiating lipid oxidation. The enzymatic systems that reduce iron in the membrane were reported to be very important because they generate active catalysts in the presence of highly polyunsaturated lipids [46]. Thus, the main systems involved in the initiation process of lipid oxidation in meat are microsomal enzymes peroxidases and dioxygenases [46,65].

The main enzyme that initiates lipid oxidation is lipoxygenase. This enzyme is present in animal tissues and it is essential for the cell membrane biosynthesis of some long-chain fatty acids [2]. Various studies reported that lipoxygenase can abstract hydrogen from the allylic methylene position of polyunsaturated fatty acid, resulting in a conjugated diene hydroperoxy [7]. Additionally, lipoxygenase can directly oxygenate polyunsaturated fatty acids, even when lipids are bound to membranes of lipoproteins, forming lipid hydroperoxides [4]. Thus, this enzyme is directly involved in the initiation phase of lipid oxidation [2,65].

Another important prooxidant enzyme that remains present in the meat after animal slaughter is the myeloperoxidase. This enzyme is found in the blood cells, and normally the meat is contaminated by contact with the blood during slaughter. Residual concentrations of the enzyme with prooxidant activity remaining even after blood washing [46]. Myeloperoxidase catalyses the reaction between H_2_O_2_ and chloride resulting in hypochlorous acid that reacts with O_2_•^−^ giving rise to the OH• [98]. The importance of this enzyme is that the reaction that it catalyses is six times faster than the Fenton reaction, does not need the presence of metals and produces the most important radical in the initiation of oxidation [7].

#### 3.1.5. Endogenous Antioxidants

In addition to all prooxidant factors that have been mentioned, there are also antioxidant compounds that protect the meat from the action of free radicals or catalysts that promote lipid oxidation. These endogenous compounds can be subdivided into three main groups: vitamins, peptides and enzymes. The mechanism of actions of these antioxidants is mainly due to their ability to neutralize radicals (both, reactive oxygen species and hydroperoxide radicals) or scavenger metals that catalyse the oxidation process. 

##### (a) Vitamins

One of the most effective antioxidant vitamins present in animal tissues is α-tocopherol (Vitamin E). It is a lipid-soluble vitamin and is preferentially incorporated into lipid membranes (phospholipids) [99]. The fact that it is located in phospholipids means its content is highly influenced by the type of muscle. Oxidative muscles, which have a greater number of cell membranes, show a greater amount of this vitamin than glycolytic muscles [46]. The antioxidant activity is concentration dependent, thus meat that contains high amounts of α-tocopherol has greater oxidative stability [6]. The main antioxidant activity of α-tocopherol can be attributed to it competing with polyunsaturated fatty acids to donate a hydrogen. Lipid radicals attack α-tocopherol much faster than lipids, therefore, unsaturated fatty acids are protected from the action of these radicals. During this reaction, α-tocopherol transfers a hydrogen atom to lipid peroxy radical and scavenges the peroxy radicals, resulting in a tocopheroxy radical that is more stable than other radicals due to resonance structures [50]. Additionally, α-tocopherol also delays the decomposition of hydroperoxides [100], scavengers ^1^O_2_ and complexes metals in the presence of ascorbate [46], which inhibits the propagation phase of lipid oxidation.

Subsequent reactions of the tocopheroxy radicals will depend on the level of oxidation. At a low oxidation level, the tocopheroxy radicals react with each other giving rise to tocopherol and tocopheryl quinone, while at a high oxidation level tocopheroxy radical reacts with peroxy radicals to produce a complex that can be subsequently hydrolysed giving rise to hydroperoxide and tocopheryl quinone [50]. Another way to neutralize tocopheroxy radicals is that they react with ascorbic acid at the lipid/water interface, regenerating the α-tocopherol molecule [6,95].

On the other hand, within lipid-soluble vitamins, the content of retinol (vitamin A) and carotenes (pro-vitamin A) also plays an important role in the oxidative stability of meat. Similar to α-tocopherol, carotenoids protect polyunsaturated fatty acids from oxidation through scavenging peroxy radicals, resulting in a carbon-centered radical that is stabilized by resonance [46].

Finally, ascorbic acid (vitamin C) is a water–soluble vitamin that also may act as an endogenous antioxidant. As commented above, it plays a very important antioxidant role in restoring α-tocopherol from the tocopheroxy radical [95]. Furthermore, ascorbic acid can scavenge oxygen and various free radicals (O_2_•^−^, OH• or OOH•) [7], and also maintain heme-proteins in a reduced non-catalytic form [46,101]. 

##### (b) Peptides

Some meat peptides are recognized as antioxidant compounds, so they increase the oxidative stability of meat [95]. In fact, a study has concluded that the meat stability was positively influenced by the content of antioxidant peptides [102]. The mechanism of the action of peptides as antioxidants is not fully understood, although it is clear that they scavenge radicals, reduce hydroperoxides and chelate metals. The antioxidant activity of the peptides depends largely on the amino acids they contain in their chemical structure [103]. 

Most of the peptides that show antioxidant activity present valine or leucine in the amino-terminal position and have also proline, histidine, tyrosine, tryptophan, methionine and/or cysteine in their amino acid sequence. In this regard, peptides with tryptophan or tyrosine in carbonyl-terminal position are capable to scavenger radicals and some reactive oxygen species, while those that have histidine in their composition are able to chelate metals like iron or copper [52,104].

Carnosine (β-alanyl-l-histidine) and anserine (β-alanyl-*N*-methyl-histidine) are known to be the most abundant antioxidant endogenous dipeptides and they are present in meat at high amounts [52]. Both dipeptides are capable of chelating transition metals and scavenging peroxy radicals [46]. In addition, these dipeptides and other compounds such histidine, lysine, albumin, and sulphur/amine compounds can trap aldehydes, reducing rancidity and the catalysis of the heme-protein oxidation [52,105].

Regarding the antioxidant activity of carnosine and anserine, there is some controversy. Multiple studies have proven its protective efficacy in several model systems [106,107]. However, more recent studies state that these peptides are not capable of delaying oxidation [108,109,110]. Therefore, the role that these peptides play in the oxidative stability of meat is not yet fully demonstrated. 

Another antioxidant peptide is glutathione, which acts as an antioxidant in various pathways. One of these ways is that glutathione reduces reactive ferryl heme-protein to met heme-protein [111]. Moreover, glutathione can produce a reduction of hydroxyl, peroxy or alkoxy radicals to hydroperoxides and glutathione disulphide [112]. However, during this reaction O_2_•^−^ can be produced. So, if this reactive species is not removed, the antioxidant activity of glutathione may be minimal [46].

##### (c) Enzymes

Although to the aforementioned prooxidant enzymes, there are endogenous enzymes with antioxidant activity. These are mainly superoxide dismutase, catalase and glutathione peroxidase. The antioxidant activity of the superoxide dismutase is due to this enzyme removing O_2_•^−^ and producing oxygen and H_2_O_2_ [7]. Additionally, the H_2_O_2_ formed during the activity of the superoxide dismutase can be eliminated by catalase, which transforms them into water and molecular oxygen [7,52]. 

On the other hand, glutathione peroxidase prevents lipid oxidation in different ways. Similar to catalase, glutathione peroxidase also removes H_2_O_2_ resulting water and oxidized glutathione. Furthermore, the hydroperoxides could be reduced by this enzyme activity, resulting in the formation of alcohol, water and oxidized glutathione [6,52,113].

### 3.2. Storage Conditions

#### 3.2.1. Time and Temperature

Knowing that oxidative processes are multiple chemical reactions, it is clear that like any other chemical reaction, oxidation is favoured as both a time and temperature increase [6,114].

As expected, the contact between the substrates and the oxidation catalyst is favoured by the temperature. In addition, it is necessary to take into account the decomposition of hydroperoxides which also increase with increasing temperature [50], promoting the propagation phase. However, not only the temperature value is decisive, but in frozen products the freezing process and the possible temperature fluctuations that it suffers are very important. This is due to the fact that the temperature fluctuations cause the formation of extracellular ice crystals, which increases cell disruption, releasing prooxidant compounds and promoting oxidation [46,115]. Meat subjected to the frozen-thawed process suffers from an accelerated lipid oxidation that starts in the cellular membrane to continue with radical secondary lipid oxidation during thawing [116,117]. The freezing temperature and the rate of thawing are important factors, which is going to affect the meat quality. To this regard, freezing processes at low temperatures cause less cell damage because the freezing process is faster and therefore the ice crystals are smaller [7]. With regards to thawing, a reduction in the thawing time results in the formation of less exudate and water holding capacity, which means a decrease in the disruption of the muscle fibre structure [118].

On the other hand, storage time also influences the oxidation processes. The possibility that radicals cause damage to lipids increases with time. To this we must add that long storage periods promote the release of iron from heme-proteins [7], which catalyse multiple reactions in the initiation and propagation phases of lipid oxidation.

#### 3.2.2. Packaging and O_2_ Concentration

Modern packaging methods offer advantages over conventional techniques. In meat and meat products the most common techniques are vacuum packaging, modified atmosphere packaging and active packaging [52]. However, all of them have advantages and disadvantages depending on the product which is being preserved. First of all, it should be noted that the gas composition of the atmosphere surrounding the product is essential for the development of the oxidative processes. From the point of view of lipid oxidation, taking into account that oxygen acts as the main component of the propagation phase through its ability to react with alkyl radicals to form peroxy radicals and also as a source of reactive oxygen species [7], it seems clear that reducing its concentration in the package would be a good strategy to limit the oxidation of meat [2]. This aspect was confirmed by different authors who found lower lipid oxidation in meat packaged in low O_2_ concentration or vacuum packed, than those packaged in atmospheres containing high O_2_ amounts [119,120]. 

One of the easiest ways to reduce oxygen concentration is the use of nitrogen-based atmosphere packaging or vacuum-packaging. However, under certain circumstances it is not possible to eliminate oxygen. The clearest case is in fresh meat, especially red meat, which must be packaged in a rich O_2_ atmosphere to maintain the desirable red colour of the meat. The main drawback to using high O_2_ amounts is that rancidity develops even when the colour is still desirable [52]. 

On the other hand, not only the presence of oxygen influences lipid oxidation reactions. As commented above, light is another important parameter to control in order to increase the oxidative stability of meat, since it promotes the initiation phase through photo-oxidation and the production of reactive ^1^O_2_. Therefore, the use of packaging materials that absorb UV light prevent photo-oxidation and increases the oxidative stability [46]. 

Nowadays, the use of active packaging provides alternatives that allow the control of oxidation more efficiently. Recently implemented strategies involve the use of films containing antioxidants, which allows an increased shelf life of meat and meat products during storage [9]. Therefore, taking into account the product to be packaged (fresh meat or meat product), the correct choice of the package and packaging material, the technology that will be used to prevent oxidation and also the presentation format is vital in minimizing quality loss [9,52].

## 4. Methods for the Measurement of Compounds Derived from Lipid Oxidation 

As mentioned during this review, the wide variety of factors that influence oxidation, different compositions of meat and meat products, as well as the complexity of reactions and interactions during lipid oxidation make it practically impossible to develop a unique technique to measure the degree of oxidation. However, taking into account the importance that oxidation has on product quality, it is necessary to measure the level of oxidation in order to establish strategies to minimize quality losses. For this reason, there are a variety of analytical techniques that help in the identification and quantification of changes in reagents and products of lipid oxidation. 

As commented above, hydroperoxides are the most important initial oxidation products, which are rapidly decomposed due to their transitory nature giving rise to secondary compounds [17]. Therefore, lipid oxidation methods could measure changes in primary products (changes in fatty acids and formation of lipid hydroperoxides and conjugated dienes/trienes) and changes in secondary products (formation of carbonyls, aldehydes, volatiles, malondialdehyde) [25,121]. Because the choice of a method is very difficult, the most common and adequate to obtain reliable and complete information on the state of oxidation of meat is the measure of both primary and secondary oxidation compounds.

Additionally, of all these difficulties it is necessary to add that the meat is a complex matrix that generally causes the appearance of artefacts, affecting the obtained results. So, the analyte extraction and isolation process are the main challenge to ensure a specific, sensitive, reproducible and rapid analysis, with the objective of having objective and accurate results. 

The present review describes routine methods to determine lipid oxidation products in meat and meat products. Table 3 show typical values of specific oxidation compounds obtained from different meat products. More recently, other analytical techniques have been developed (chemiluminescence, fluorescence emission, Raman spectroscopy, infrared spectroscopy or magnetic resonance). However, these techniques, which were reviewed by other authors [49,122,123], are normally used to monitor oxidation under very specific conditions, and are not easily adapted methods to the laboratory routine. In addition, both the meticulous experimental work and the interpretation of the results require very specific knowledge. They are also laborious and need expensive equipment. So, these techniques were not covered by this review.

### 4.1. Compounds Derived from the Primary Changes

The main methods that analyse the primary changes during lipid oxidation can be subdivided into those that measure the loss of reactants and those that measure the production of the primary compounds derived from oxidation [48].

#### 4.1.1. Changes in Substrates

A significant decrease in the content of unsaturated fatty acids is expected during lipid oxidation, because they are the main substrate for oxidative reactions. Therefore, observing the changes produced in the content of these fatty acids would allow us to monitor the evolution of lipid oxidation [52].

However, this technique is not usually used in assessing lipid oxidation. This is due to several factors. It first requires total fat extraction, subsequent conversion to derivatives and analysis normally by gas chromatography. Because total lipids (triglycerides and phospholipids) must be extracted, solvent selection is a critical point in this analysis [123]. In addition, taking into account that lipid oxidation begins in the phospholipid fraction, the separation of phospholipids from the other lipids is also usually a necessary step to obtain accurate results [121]. Another important point by which the measurement of fatty acid composition is not a good indicator to monitor oxidation is that an effective loss of unsaturated fatty acids can only be quantified at the latest stage of the oxidation process [28]. For this reason, another more specific type of technique is usually performed, such as the measure of the production of primary products [48]. The analysis of fatty acids is therefore a complementary indicator of the degree of oxidation.

#### 4.1.2. Peroxides

The primary products of oxidation are lipid hydroperoxides. The measurement of hydroperoxides production, also called peroxide value, has long been used as the main indicator of the production of primary oxidation compounds in meat and meat products. 

During the early stages of oxidation, an increase in hydroperoxides is observed, because the formation level is higher than that of decomposition. However, because these compounds are unstable, in more advanced stages of oxidation, the process of decomposition of hydroperoxides is greater than that of formation, so a decrease in the content of hydroperoxides (peroxide value) is observed. Thus, a low peroxide value may represent both, early or advanced oxidation [17]. With this in mind it is easy to conclude that in advanced stages of oxidation the use of peroxides as an oxidation indicator would result in an underestimation of the degree of oxidation [25], so it would not be a good indicator in highly oxidized meat [121]. In this regard, despite peroxide value being a widely used parameter to know the degree of oxidation, this is only effective in the initial stages of oxidative processes [123]. In fact, it is recommended to monitor the evolution of the peroxide value over time to have complete information on the state of lipid oxidation [122,123], and know if the lipid is in the growth or decay portion of the hydroperoxide concentration curve [123].

The methods used for the determination of peroxides are based on their reducing power [49]. These require a prior extraction of lipids [48] in which hydroperoxides are found, which easily oxidize inorganic ions such as iodine or iron. The two main techniques used for the determination of peroxides are detailed below.

##### (a) Iodometric Titration

Iodometric titration is the method most commonly used to measure the peroxide value due mainly to the simplicity of the experimental procedure. As its name suggests, this method is based on the titration of iodine released during the reaction with hydroperoxides [17]. To this regard, iodide ion (I^−^) added as potassium iodide reduces hydroperoxides in acidic medium resulting in iodine (I_2_). The concentration of hydroperoxides is therefore directly proportional to the release of I_2_, which is measured by titration with sodium thiosulfate in the presence of starch that acts as an indicator [123]. The results of peroxide value is normally expressed as milliequivalents of oxygen/kg lipid [17].

However, this method has a number of drawbacks that can lead to erroneous results. The main sources of error are due to the fact that I_2_ can be produced by oxidation of the potassium iodide in the presence of oxygen and also that the released iodine can be absorbed at unsaturation sites of fatty acids [49]. Additionally, the results can also be influenced by the temperatures and reaction time, and by the structure and reactivity of the peroxides [123]. In spite of these drawbacks, the use of this method together with the determination of some secondary compound is still useful to monitor oxidation in meat and meat products [17].

##### (b) Ferric-xylenol Orange (FOX)

The use of ferrous oxidation as a method for the determination of peroxide value was proposed since it is a simpler method to use than iodometric titration, and ferrous ion is much less susceptible to spontaneous oxidation with the atmospheric oxygen during the reaction than the iodine [49].

In a similar way to iodometric titration, the ferrous ion reduces hydroperoxides in acidic conditions resulting in ferric ion. The ferric ion reacts with xylenol orange that acts as an indicator and produces a complex with a maximum absorbance between 550–600 nm [49]. Thus, the hydroperoxides amount, which is directly related with ferric ion concentration, could be measured with a UV-Vis spectrophotometer. This method is simple, fast and as commented above, it does not depend on the presence of oxygen [17]. Moreover, different authors point out that FOX is a very high sensitive method for the determination of hydroperoxides [130] and that the FOX results are correlated better with other oxidation parameters than iodometric titration [131]. 

#### 4.1.3. Conjugated Compounds (Dienes and Trienes) 

As commented in the “mechanism of lipid oxidation” section, the alkyl radical produced during the initiation stage tends to be stabilized by a double-bound rearrangement to form a conjugated dienes or trienes [49]. Therefore, the abnormal production of conjugated compounds can be used to measure lipid oxidation in meat because their increases are proportional to the formation of hydroperoxides [4]. However, as it occurs in peroxide value methods, the measurement of conjugated compounds is only effective at low oxidation stages in which hydroperoxides undergo low decomposition.

The measurement of conjugated compounds has been a method used to monitor oxidation in meat and meat products, since it is a very simple method, needs small sample amounts and does not require any reaction or reagents. This determination is carried out in meat by prior extraction of the conjugated compounds with a small amount of organic solvent, normally a mixture of hexane/isopropanol or chloroform/methanol. Then the conjugated dienes and trienes concentration is measured directly in the organic phase at 234 and 268 nm, respectively in a UV-Vis spectrophotometer [17,123]. 

The conjugated compound measurement is faster and simpler than the peroxide value. Furthermore, it does not depend on chemical reactions [123]. However, a number of drawbacks make this determination undesirable to determine oxidation under certain conditions. In fact, it was found that the method of conjugated compounds is a much less sensitive and specific measure than the peroxide value methods. The result in conjugated compound measurement could be interfered by compounds absorbing in the same region as the presence of conjugated double bonds in the original fatty acids [17] or the presence of carotenoids [122]. Moreover, the oleic hydroperoxides cannot be detected because they only contain a double bond, so the level of lipid oxidation may be underestimated [49]. Therefore, the use of other methods together with conjugated compounds measurement is recommended for proper monitoring of lipid oxidation [122]. 

#### 4.1.4. Cholesterol Oxidation Products

GC and HPLC are the most widely used techniques for the analysis of COPs. GC–MS is the most accurate and commonly applied quantification method for these kinds of compounds, giving highly promising results [132,133]. The experimental procedure involves lipid extraction, saponification, purification and derivatization in order to enhance their volatility and thermal stability and to reduce contaminants (traces of cholesterol and/or partial glycerides) [134]. Satisfactory resolution, good repeatability and sensitivity make it a valid technique for the quantification of COPs in real samples [94,132,135]. The limits of detection (LODs) and limits of quantification (LOQs) are in the range of 0.02–47.07 ng/g and 0.06–156.90 ng/g [135].

In contrast, UPLC-MS/MS was considered interesting to the determination of the profile of the main COPs in widely consumed foodstuffs since it provides reliable results in a short amount of time with low detection limits. This technique allows chromatographic run time to decrease to 4.2 min per sample, while the use of MS/MS with triple quadrupole confirms the identification of COPs detected in samples. The LOD and LOQ values range from 0.03 tο 0.16 μg/250 mg and 0.12 to 0.49 μg/250 mg fat, respectively [136].

### 4.2. Compounds Derived from the Secondary Changes

As previously mentioned, the measurement of the compounds derived from the first changes of oxidation gives reliable information on the oxidation state only during the early phases. This is mainly due to the fact that primary products are so unstable, and they decompose rapidly decreasing their content as oxidation increases. So, highly oxidized products would not be good indicators. For this reason, the measurement of secondary compounds is usually more appropriate for the determination of oxidation level of meat or meat products [48]. The secondary compounds are stable and are responsible for the appearance of rancid flavours and odours. Therefore, they are of great importance because they reflect the consequences that oxidative reactions produce on the deterioration of meat quality [17].

#### 4.2.1. TBARs

Malondialdehyde or MDA (1,3-propanedial) is one of the most important aldehydes produced during the secondary lipid oxidation of polyunsaturated fatty acids. This aldehyde also has great importance in meat because at low amounts it produces rancid aromas [137], and it is considered to be the major marker of lipid oxidation [10]. In fact, different studies established values of 2–2.5 mg MDA/kg as the accepted limit in which there is no rancidity in meat and meat products [138,139].

Several methods have been proposed for the determination of MDA. This is because it is a highly reactive molecule that is normally bound to other food ingredients [10,17]. Thus, it must be conveniently released before measurement so that it can react with the reagents. To release the bound MDA, acid and/or heat treatments were proposed [17].

The thiobarbituric acid test (TBA) is the main technique to quantify MDA. It consists of a colorimetric measure of a complex formed between TBA and MDA. However, although MDA is the major TBA reactive substance, it is well known that the reaction with TBA is not specific for MDA, but there are multiple aldehydes and other oxidation products that also react with TBA. Consequently, the method is called thiobarbituric acid reactive substances (TBARs) in order to encompass all substances that react with the TBA [17,122].

Before the measurement of TBARs, a complete extraction of the MDA must be performed. To this regard, different procedures have been proposed to perform the extraction in meat and meat products, being the most common distillation and acid extraction methods [17].

The acid extraction method consists of the homogenization of meat, usually with trichloroacetic acid, followed by centrifugation to obtain the extract [49]. This method is relatively simple, nevertheless it has an important drawback since it extracts coloured components (pigments and colourants) and solubilizes proteins, resulting in coloured or turbid extracts that may cause overestimation of TBARs value [121]. Moreover, the extraction of some ingredients that react with MDA and block the reaction with TBA results in an underestimation of the value of TBARs [137]. In fact, in cured meats the residual nitrites could produce the nitridation of MDA [48].

The distillation method avoids the problems derived from the extraction with acid, since with distillation no interfering substances are extracted. The volatile compounds derived from oxidation are separated from the meat with an appropriate solvent followed by the distillation step. This distilled extract is used for the TBARs measurement [137]. The main drawback of this method is the fact that the application of high temperatures for distillation favours the degradation of existing hydroperoxides and new thiobarbituric reactive substances can be formed which would lead to an overestimation of the TBARs value [17]. Therefore, the main disadvantage of both methods is the possibility of artefact formation [123].

After the extraction procedures, the reaction occurs when two moles of TBA react with one mole of MDA and produced a red complex. This reaction is carried out at low pH levels and high temperatures (around 90–100 °C) [49]. The coloured complex formed offers a maximum absorbance at 532–535 nm. Thus, the quantification of MDA is done with the UV-Vis spectrophotometer. 

On the other hand, due to the fact that TBA is not specific for MDA and may react with other compounds, it was proposed that TBARs procedure was used to assess the general lipid oxidation, rather than the quantification of MDA [48,137]. So, TBARs test is the most widely used for estimation of the oxidative state of meat and meat products, due to it being simple and correlated well with the sensory deterioration of meat [121]. 

More recently, chromatographic techniques have been developed in order to quantify the MDA content. These normally include the separation of MDA adduct by reverse-phase liquid chromatography and detection by photodiode array (PAD) or fluorescence. In a recent study, authors validate a UPLC method for the quantification of MDA-TBA_2_ complex, which are detected by PAD (530 nm) and fluorescence (λ_ex_ = 530 nm / λ_em_ = 550 nm) in multiple raw meat and meat products [140]. In other cases, the MDA is derivatized with 2,4-dinitrophenylhydrazine to form dinitrophenylhydrozone derivatives that are quantifiable by PAD at 300–330 nm [49]. GC methods to quantify MDA have also been reported, however the LC is more used [123]. 

It is important to mention that although chromatographic techniques offer better specificity and sensitivity for the quantification of MDA, spectrophotometric methods are preferable during routine analyses due to their simplicity and low cost [10].

#### 4.2.2. *p*-Anisidine

The decomposition of lipid hydroperoxides, apart from MDA, generates multiple aldehydes including alkanals, 2-alkenals and 2,4-alkadienals. The *p*-anisidine is a common spectroscopic method for the measure of secondary lipid oxidation which quantifies carbonyls, especially unsaturated aldehydes [122]. The reaction is based on the reactivity of the aldehyde carbonyl bond on the *p*-anisidine amine group. This reaction produces a Schiff base that reaches a maximum absorbance at 350 nm [137]. 

Although a lipid extraction is necessary before performing the analysis in meat and meat products, *p*-anisidine value is a good indicator since it shows a good correlation with other indicators of both primary (peroxide value) and secondary oxidation (TBARs and volatile aldehydes) as well as with the deterioration of organoleptic quality [49].

#### 4.2.3. TOTOX

The analyse of a single oxidation product is usually not recommended because it does not offer complete and reliable information on the oxidation state of a sample. A combination of primary and secondary lipid oxidation measurements is more convenient because they offer information about the entire oxidation process (secondary products) and the current oxidative status (primary products). With this in mind, despite not being an analytical technique itself, it was proposed to use the total oxidation index (TOTOX) to calculate the total oxidation of the meat samples. This index is calculated by combining the values of *p*-anisidine and peroxide values (TOTOX = 2 Peroxide Value + ρ-anisidine) [137]. The proposed formula is due to the fact that an increase of one peroxide value unit corresponds to an increase of two units of *p*-anisidine [123]. Lipids with Totox value below 10 are considered to be of good quality [121]. Some scientists also suggested the use of total oxidation taking into account the value of TBARs instead of *p*-anisidine (TOTOX _TBARs_ = 2 Peroxide Value + TBARs). However, this modification of the Totox index needs to be studied more in depth and validated to ensure that the results are as reliable as with the “original” equation [137].

#### 4.2.4. Carbonyls

The measurement of carbonyls (aldehydes and ketones) in meat samples also allows us to monitor secondary oxidation processes. To make this determination, the sample is reacted with 2,4-dinitrophenylhydrazine in aqueous medium. This converts the carbonyls into orange-coloured hydrazones, which are extracted with hexane and spectrophotometrically measured at 340 nm [121]. This is a simple and fast method that is well correlated with rancid flavour deterioration [53].

Other researches also measure the individual carbonyls, using liquid chromatography. In hams, 2,4-dinitrophenylhydrazones derivatives of several aldehydes including hexanal, heptanal, octanal, nonanal, 2-nonenal, 2,4-nonadienal and 2,4-decadienal were analysed using reversed-phase HPLC and detected them at 360 nm using a photo-diode array detector [141]. However, the use of this chromatographic technique, although it is much more specific, implies the need for much more expensive equipment and specialized personnel, so the spectrophotometric technique is still most commonly used. 

#### 4.2.5. Volatile Compounds

As discussed throughout this review, the process of lipid oxidation involves complex reactions that can occur in different pathways, thus giving rise to a wide variety of compounds. The secondary products of oxidation include volatile compounds with different functional groups such as aldehydes, ketones, alcohols, carboxylic acids and hydrocarbons [129,142,143,144]. Some of the volatile compounds derived from oleic, linoleic and linolenic acids are summarized in Table 4. 

The volatile character of these compounds determines their great role in the appearance of rancid flavours and odours [122]. Therefore, the determination of volatile allows us to detect several compounds derived from multiple lipid oxidation processes, avoiding part of the problems of the aforementioned analytical techniques.

Within the volatile compounds derived from lipid oxidation, aldehydes are the most abundant [41,145,146,147,148]. Among aldehydes, hexanal has been considered to be the greatest indicator of lipid oxidation in meat and meat products for years, since its content increases to a greater extent than that of other aldehydes. However, researches verify that some of these volatile compounds are highly specific to the oxidation of particular fatty acids: hexanal and pentanal are very good indicators for meats and meat products rich in n-6 polyunsaturated fatty acids, while in those that contain large amounts of n-3 polyunsaturated fatty acids, propanal could be a better indicator [49]. The content of these aldehydes present a strong correlation with TBARs and sensory scores, which confirms the great utility of these compounds when used as markers of lipid oxidation [25,48]. Other aldehydes including propenal, 4-heptenal, 2,4-heptadienal, 2-octenal, 2-nonenal or 4-hydroxy-2- trans-nonenal may also be used as indicators, but usually saturated aldehydes (propanal, pentanal and hexanal) are used because they are much more stable than unsaturated ones [49]. However, measuring the degree of oxidation with one or two markers is an erroneous approach considering that volatile determination techniques allow the simultaneous detection and quantification of several compounds, which give us more realistic and complete information. Therefore, the determination of specific compounds derived from the hydroperoxide decomposition may be a more accurate method than the measure of TBARs or total carbonyls. 

Several analytical methods have been proposed for separation, identification and quantification of lipid-derived volatile compounds. Of all of them, the use of gas chromatography coupled to mass spectrometry (GC-MS) is postulated as the preferred option [17,25,122]. In fact, it is the most widely used separation technique for the determination of volatile compounds, since it allows for a screening of all volatile compounds that integrate a meat sample (full-scan mode) or specifically selecting the markers to be used (single-ion monitoring mode and also full-scan mode). Moreover, the possibility of using database and libraries containing the spectra of most compounds of interest allows a quick and reliable identification of all of them. 

Despite GC-MS being a powerful tool to detect and quantify all compounds derived from lipid oxidation, it is useless if sampling is not prepared well. In fact, a critical point is the extraction and recovery procedure of volatiles since new oxidation products can be generated or also degrade the existing compounds [25]. To this regard, the most widely used methods for the extraction of volatiles are solvent extraction/distillation and headspace techniques [49].

One method for the extraction of volatile compounds from meat and meat products is the simultaneous steam distillation with solvent extraction. In this case, the samples are distilled with an appropriated solvent for several hours. Then, the solvent is removed by evaporation. This method recovers high amounts of volatiles, however it has been criticized because the high temperatures used in the distillation process could promote the generation of new lipid oxidation compounds and the evaporation of solvent may result in the loss of volatile compounds [25]. Another similar technique is the reduced pressure steam distillation extraction that allows evaporation of the sample at a much lower temperature than simultaneous distillation extraction, thus reducing the possibility of forming artefacts [49].

On the other hand, the headspace extraction procedures could be subdivided into three main methods. An important advantage of these methods is the absence of solvent peak during the analysis. 

The first headspace method is static headspace. In this extraction procedure, the meat sample is placed in a sealed vial and allowed to stand to establish equilibrium, at a controlled temperature, between the sample and headspace. Therefore, the volatile pass from the sample to headspace and an aliquot is injected on the GC column [25]. It is simple, does not require solvent extractions and may be automated. However, this method can only quantify a low fraction of the compounds of interest resulting in low sample recovery and limiting the sensitivity [25]. With the objective of increasing the recovery of certain compounds, we can increase the extraction temperature, but this compromises the stability of the compounds and can change their concentration and for this reason it is not advisable [49].

To overcome this problem, the dynamic headspace technique was developed. This method does not require an equilibrium, since the sample is continually purged by inert gas to extract volatile compounds, which are trapped in porous polymer [49]. This allows the extraction and concentration of a large part of the compounds, which will be subsequently injected into the GC resulting in a greater sensitivity. However, the main drawbacks of this technique are that the instrumentation requires the monitoring of several steps which increases the sources of error, the necessary instrumentation is much more complex and expensive and is not easily applicable to a large number of samples [49]. 

Finally, the other headspace technique is the use of solid phase microextraction or SPME. In a similar way to the static headspace, the volatile pass reaches an equilibrium between the sample and the headspace at a selected temperature. However, in SPME, the headspace volatiles are adsorbed onto a fused silica fibre, coated with a specific polymer. This process concentrates the target compounds that are then desorbed into the GC injection port [24]. Nonetheless, SPME is a technique especially sensitive to small changes produced in any of the factors involved in the extraction. The results are altered through the use of different coating fibre, time or temperature of adsorption and desorption [149]. Moreover, rapid deterioration of fibre coating could also influence the results [25]. Although these precautions must be taken into account, SPME is a recognized technique for the analysis of the volatile due to it being simple, not requiring solvents, being easily automated and having very high sensitivity [150]. Among the techniques for the analysis of lipid-derived volatiles, the use of SPME is growing in popularity due to its sensitivity, ease of use and it providing a lot of information about several compounds derived from lipid oxidation. 

## 5. Conclusions

Meat and meat products are very complex matrices with a composition that makes them susceptible to the oxidation processes. In fact, the oxidative processes on lipids, proteins, pigments and vitamins are frequent and interrelated, negatively affecting the quality of the meat, including colour and texture changes, rancidity development, nutrient losses and the formation of toxic compounds.

On the other hand, despite the fact that lipid oxidation has been widely investigated for decades, the enormous complexity of the reactions as well as the different pathways, the formation of unstable compounds that are rapidly degraded and the multiple factors that affect the oxidative reactions mean that mechanisms involved in lipid oxidation have not yet been completely understood. Therefore, this review aims to summarize the main mechanisms involved in the oxidative processes, the influence that meat composition and storage conditions exert on these reactions as well as the main routine analytical methods to determine the degree of oxidation of meat and meat products.

The information presented in the reviews allows an understanding of the limitations and advantages of existing methods, which helps to make the decision of which is the most appropriate for each case, taking into account the compounds to be analysed, the meat or meat product to analyse and the experimental conditions that are required. Therefore, taking into account that each method gives detailed and specific information on only a part of the oxidative processes it seems clear that a combination of various methods is necessary to determine the oxidation state of the sample. As a general rule, lipid oxidation methods should have a good correlation with sensory scores, since organoleptic changes significantly affect meat quality.

## Figures and Tables

**Table 1:** Fat and fatty acid contents of different animal sources.

Lipid Composition	Animal Source					
	Beef	Chicken	Deer	Foal	Pig	Turkey
Fat (%)	0.75–2.80	0.10–2.49	0.05–0.34	0.15–0.67	2.00–3.24	0.38–4.24
*Fatty acids (g/100 g of fatty acids)*						
C14:0	1.47–3.01	0.46–0.87	0.81–3.16	1.19–2.78	1.35–1.50	1.18–1.57
C14:1	0.65–1.21	−	0.07–1.06	−	0.06–0.24	0.24–0.32
C15	0.27–0.35	−	0.30–0.52	4.14–6.70	−	0.18–0.24
C16:0	21.70–24.82	21.32–27.57	12.73–19.13	21.18–24.17	23.92–25.07	22.3–23.9
C16:1n-7	2.77–3.66	1.48–4.47	1.08–4.22	1.85–3.42	2.04–2.76	2.67–5.05
C17:0	0.99–1.29	−	0.45–0.57	1.30–2.27	0.16–0.31	0.33–0.48
C17:1n-7	0.88–1.07	−	−	1.15–1.66	0.16–0.24	0.32–0.55
C18:0	10.49–14.51	7.13–10.40	14.40–16.01	6.02–7.28	12.48–13.75	9.0–11.0
11t–C18:1	2.96–6.18	−	0.50–0.57	−	−	0.75–1.12
C18:1n-9	30.05–32.21	26.66–37.50	11.83–14.84	10.16–14.44	35.82–44.10	30.4–33.4
C18:1n-7	1.67–2.28	1.86–2.71	1.91–2.33	−	2.82–3.23	1.96–2.34
C18:2n-6	9.11–14.33	15.68–21.27	18.22–23.57	18.68–25.79	8.49–13.92	18.6–19.9
C20:1n-9	0.16–0.17	−	−	0.04–0.25	0.70–0.93	0.27–0.34
C18:3n-3	0.25–0.34	0.48–2.93	2.50–3.73	11.10–17.37	0.41–0.68	0.90–1.22
C20:2n-6	0.08–0.14	−	−	0.32–0.39	0.40–0.59	0.20–0.25
C20:3n-6	0.58–1.38	0.27–0.56	0.77–1.27	0.64–1.10	0.08–0.13	0.17–0.35
C20:4n-6	2.28–5.50	2.10–10.91	9.06–10.74	4.26–6.44	0.66–1.83	1.36–3.82
C20:5n-3	0.14–0.24	−	1.55–2.98	1.03–1.86	−	0.10–0.21
C22:5n-6	−	−	1.71–2.09	−	−	−
C22:5n-3	0.27–0.64	0.21–2.44	2.62–3.97	−	−	0.19–0.62
C22:6n-3	−	0.29–2.36	0.57–0.97	0.70–1.25	−	0.29–0.93
SFA	35.24–44.09	31.89–35.65	30.41–38.21	37.93–40.56	38.39–41.07	34.8–36.4
MUFA	41.51–44.49	33.40–43.23	15.28–22.73	13.51–18.75	41.92–51.22	37.4–42.8
PUFA	12.80–23.07	19.78–33.84	37.55–50.05	40.68–48.14	10.39–17.01	22.3–27.5
n-3	0.65–1.28	1.30–6.59	30.16–38.22	14.52–19.86	0.41–0.68	20.6–24.5
n-6	11.92–21.59	18.46–27.71	7.23–11.66	23.59–32.73	9.79–16.81	1.83–2.88
n-6/n-3	15.73–18.61	4.34–15.80	3.54–4.33	1.36–2.32	23.88–24.72	8.88–12.3

SFA: Saturated fatty acids; MUFA: Monounsaturated fatty acids; PUFA: Polyunsaturated fatty acids; Data were obtained from [62,66,67,68,69,70].

**Table 2:** Fat and fatty acid contents of typical pork meat products.

Lipid Composition	Meat Product					
	Chorizo	Ham	Lacón	Loin	Pâté	Salchichón
Fat (%)	17.7	3.7–5.2	4.0–4.7	13.3–13.9	18.23	22.5
*Fatty acids (g/100 g of fatty acids)*						
C10:0	–	–	0.14–0.18	0.04–0.05	0.04	–
C12:0	–	–	0.06–0.18	0.04–0.05	0.06	–
C14:0	2.53	1.25–1.84	1.35–1.73	1.09–1.21	1.32	1.26
C16:0	25.71	21.9–23.5	20.4–24.1	23.4–24.4	20.61	22.17
C16:1n-7	3.05	2.28–3.56	0.40–4.24	2.18–2.31	2.01	2.27
C17:0	0.84	0.31–0.40	0.20–0.33	0.09–0.14	0.26	0.36
C17:1n-7	0.53	0.29–0.39	0.23–0.38	0.11–0.17	0.25	0.26
C18:0	14.72	11.3–15.0	7.46–13.9	11.0–12.2	10.50	11.10
9t-C18:1	0.53	–	–	–	0.30	0.34
11t-C18:1	–	–	–	–	–	0.14
C18:1n-9	39.9	39.8–44.9	36.4–43.8	47.9–48.8	42.92	39.25
C18:1n-7	–	–	3.41–3.52	–	3.20	2.98
C18:2n-6	8.02	10.9–13.6	12.7–19.3	7.97–8.68	14.14	16.03
C18:3n-3	1.19	0.74–1.13	0.59–1.53	0.31–0.34	0.98	0.83
C20:0	0.08	0.20–0.22	0.66–0.95	0.03–0.07	0.21	0.17
C20:1n-9	–	1.43–1.77	0.47–0.75	0.05	1.11	0.83
C20:2n-6	0.32	0.85–1.07	0.06–0.50	0.24–0.34	0.86	0.65
C20:4n-6	–	0.36–0.65	0.61–1.99	0.91–0.95	0.66	0.48
C20:3n-3	0.08	0.17–0.27	0.16–0.33	0.03–0.06	0.22	0.13
C20:5n-3	–	–	0.35–2.81	0.01–0.02	–	–
C22:5n-3	–	–	0.11–0.32	–	0.14	–
C22:6n-3	–	–	0.09–0.35	–	–	–
SFA	45.6	35.6–40.3	30.5–39.4	37.1–38.0	33.05	35.36
MUFA	44.3	43.8–50.7	44.0–48.8	50.4–51–5	49.84	46.05
PUFA	9.94	13.7–15.8	16.5–24.4	11.1–11.6	17.12	18.60
n-3	1.82	0.98–1.40	1.05–2.11	–	1.34	1.14
n-6	14.04	11.3–13.9	15.2–20.4	–	15.78	17.34
n-6/n-3	7.71	8.88–14.2	9.89–13.3	–	11.69	15.44

SFA: Saturated fatty acids; MUFA: Monounsaturated fatty acids; PUFA: Polyunsaturated fatty acids; Data were obtained from [75,76,77,78,79,80,81].

**Table 3:** Compounds derived from lipid oxidation in meat and meat products.

Lipid Oxidation Derived Compounds	Indicator	Method	Meat Product	Oxidation Values	Reference
**Compounds from primary changes**	Peroxides	Iodometric titration	Pork pâté	3.80–10.99 meqO_2_/kg	[124]
			Dry-cured loin	16.2–20.3 meqO_2_/kg	[80]
		FOX	Beef, chicken, lamb, pork	20–40 mmol peroxides/kg meat lipid	[15]
	Conjugated compounds	Dienes UV-vis (234 nm)	Pork pâté	2.91 μmol/g	[125]
		Trienes UV-vis (268 nm)	Beef, chicken, lamb, pork	0.19–0.24 μmol/g	[15]
	COPs	GC–MS	Iberian hams	57–71 µg/100 g	[94]
**Compounds from secondary changes**	TBARs	UV-vis (532 nm)	Nuggets	0.37–1.94 mg MDA/kg	[126]
			Burgers	0.18–3.89 mg MDA/kg	[33,127]
			Spanish *Salchichón*	0.35–0.45 mg MDA/kg	[128]
			Dry-cured loin	0.17–0.30 mg MDA/kg	[80]
	Volatile compounds	SPME-GC/MS	Dry-cured loin	Pentanal: 2.3 AU × 10^4^/g Hexanal: 13.1 AU × 10^4^/g Octanal: 16.6 AU × 10^4^/g Total aldehydes: 253 AU × 10^4^/g	[24]
			Dry–cured ham	Pentanal: 63.9 AU × 10^4^/g Hexanal: 1354 AU × 10^4^/g Octanal: 73.9 AU × 10^4^/g Total aldehydes: 1990 AU × 10^4^/g	
			Salchichón	Pentanal: 13.5 AU × 10^4^/g Hexanal: 47.5 AU × 10^4^/g Octanal: 38.1 AU × 10^4^/g Total aldehydes: 345 AU × 10^4^/g	
			Dry-cured shoulder	Pentanal: 45.5 AU × 10^4^/g Hexanal: 395 AU × 10^4^/g Octanal: 41.8 AU × 10^4^/g Total aldehydes: 694 AU × 10^4^/g	
			Chorizo	Pentanal: 13.3 AU × 10^4^/g Hexanal: 287 AU × 10^4^/g Octanal: 2.5 AU × 10^4^/g Total aldehydes: 438 AU × 10^4^/g	
			Cecina	Pentanal: 59.1 AU × 10^4^/g Hexanal: 1283 AU × 10^4^/g Octanal: 140 AU × 10^4^/g Total aldehydes: 1921 AU × 10^4^/g	
			Liver pâté	Hexanal: 24.9–374 AU × 10^6^/g Heptanal: 0.94–9.34 AU × 10^6^/g Octanal: 0–5.90 AU × 10^6^/g	[124]
				Pentanal: 39.9–82.8 pg/g Hexanal: 0–156 pg/g Heptanal: 1.91–3.28 pg/g	[56]
		DHS-GC/MS	Dry-cured lacón	Pentanal: 26.6–74.9 AU × 10^6^/g Hexanal: 383–501 AU × 10^6^/g Heptanal: 17.3–33.3 AU × 10^6^/g	[129]

FOX: Ferric-xylenol orange; COPs: Cholesterol oxidation products; AU: Area units.

**Table 4:** Typical volatile compounds of oxidizing oleic, linoleic and linolenic acids.

Volatile Compounds	Oleic Acid	Linoleic Acid	Linolenic Acid
Hydrocarbons	Hexane	Butane	Ethane
	Heptane	Pentane	Butene
	Octane	1,3-Nonadiene	2-Pentene
	Nonane		3-Hexene
	1-Nonene		1,3,6-Nonatriene
	1-Decene		
Alcohols	Hexanol	Butanol	2-Pentenol
	Heptanol	Pentanol	1,3,6-Nonatrienol
	Octanol		
	Nonanol		
	1-Nonenol		
Aldehydes	Hexanal	Butanal	Propanal
	Heptanal	Pentanal	2-Pentenal
	Octanal	Hexanal	3-Hexenal
	Nonanal	3-Nonenal	2,4-Heptadienal
	Decanal	2,4-Decadienal	3,6-Nonadienal
	2-Decenal	Formaldehyde	2,4,7-Decatrienal
	2-Undecanal		Formaldehyde
	Formaldehyde		
Acids	Hexanoic acid	Octanoic acid	Octanoic acid
	Heptanoic acid	9-Undecenoic acid	9-Decenoic acid
	Octanoic acid		9-Undecenoic acid
	8-Nonenoic acid		9,11-Dodecadienoic acid
	9-Decenoic acid		

Data adapted from Schaich [45].
